# Antifungal Drug Discovery: Something Old and Something New

**DOI:** 10.1371/journal.ppat.1002870

**Published:** 2012-09-06

**Authors:** Arielle Butts, Damian J. Krysan

**Affiliations:** 1 Department of Chemistry, University of Rochester, Rochester, New York, United States of America; 2 Department of Pediatrics, University of Rochester, Rochester, New York, United States of America; 3 Department of Microbiology/Immunology, University of Rochester, Rochester, New York, United States of America; The University of North Carolina at Chapel Hill, United States of America

## We Need New Antifungal Drugs

**Figure 1:**
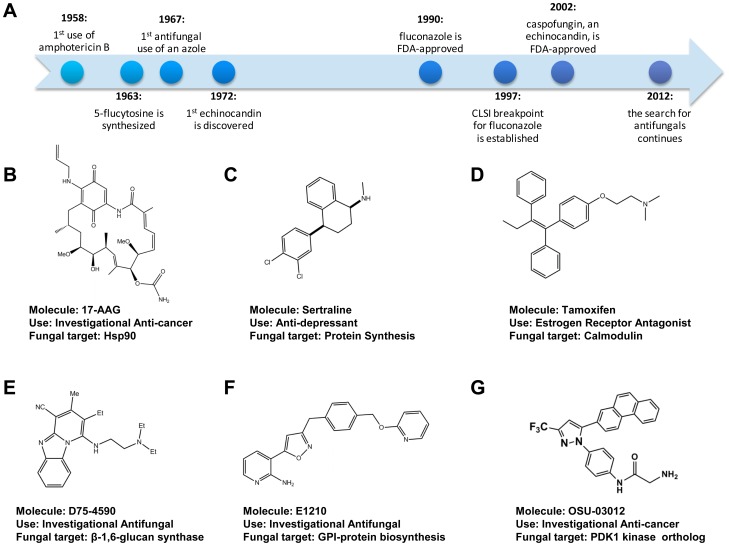
Antifungal drug development timeline. (A) Timeline of selected milestones in antifungal drug development. (B–G) Chemical structure, name, clinical use, and putative molecular target of antifungal drugs and small molecules discussed in the text.

Invasive fungal infections are devastating. Despite state-of-the-art antifungal therapy, the mortality rates for invasive infections with the three most common species of human fungal pathogens are *Candida albicans*, 20%–40% [1]; *Aspergillus fumigatus*, 50%–90% [1]; and *Cryptococcus neoformans*, 20%–70% [2]. Although invasive fungal infections can affect people with intact immune systems, the vast majority of disease occurs in the setting of an immunocompromised host. As discussed recently, the dismal outcomes for invasive fungal infections cannot be completely attributed to a lack of efficacious antifungal drugs [3]. However, because most patients with invasive fungal infections are immunocompromised, the immune system cannot effectively assist in the clearance of the infection, and consequently, the success of treatment is more dependent on the efficacy of the antifungal agent than in the setting of an immunocompetent host. Unfortunately, our repertoire of antifungal agents is limited, particularly in comparison to the number of agents available for bacterial infections. In fact, it took 30 years for the newest class of antifungal drugs, the echinocandins, to progress from bench-to-beside (Figure 1A). Furthermore, it is sobering to consider that the gold standard therapy for cryptococcal meningitis, a disease that kills more than 650,000 per year world-wide [2], is based on medications (amphotericin B and flucytosine) that were discovered nearly 50 years ago.

## New Tricks for Old Dogs: Repurposing

Recently, the concept of “re-purposing”-established medications to treat new diseases has emerged as an approach to expediting drug development in general. The toxicology and pharmacology of such compounds are established and, thus, a drug with a useful new indication could be rapidly translated to clinical use. While the antifungal activity of many “non-antifungal” drugs has been recognized for decades, interest has increased in exploring the therapeutic potential of such drugs both as single agents and as adjunctive agents in combination with established antifungal drugs.

A pioneering example of antifungal re-purposing comes from work in the Heitman lab demonstrating that clinically used calcineurin and target of rapamycin (TOR) inhibitors have antifungal activity and synergize with fluconazole [4]. More recently, the Cowen and Lindquist laboratories have shown that Hsp90 inhibitors such as the geldenamycin derivative 17-AAG (Figure 1B), developed as potential anti-cancer drugs, dramatically improve the activity of fluconazole both in vitro and in a *Galleria mellonella* model of systemic candidiasis [5]. Finally, Zhai et al. have recently reported that the combination of the anti-depressant sertraline (Figure 1C), which appears to target translation in yeast, with fluconazole decreases fungal brain burden relative to fluconazole monotherapy in a mouse model of cryptococcosis [6].

Recently, a number of groups have screened libraries of off-patent medications for agents with antifungal activity. For example, Breger et al. identified 15 compounds that prolonged survival of *C. elegans* worms infected with *C. albicans* and showed that enoxacin, a fluorquinolone antibiotic, was effective in a mouse model of disseminated candidiasis [7]. Recently, Spitzer et al. identified 148 compounds that improved the in vitro activity of fluconazole by screening a similar library against *S. cerevisiae*, *C. albicans*, *C. neoformans*, and *C. gattii* in the presence of sub-inhibitory fluconazole [8]. Although no “off-the-shelf” antifungal drugs have emerged from “repurposing” studies, the antifungal scaffolds with known pharmacological properties could serve as useful lead compounds for further development.

## New Ways of Looking: Novel Antifungal Screening Strategies

Natural products are the basis for the vast majority of anti-infective therapies in current clinical use. For example, polyenes and echinocandins, two key classes of antifungal drugs, are natural product derivatives. A challenge facing natural product-based screening is that campaigns continue to rediscover previously known molecules from new natural product collections [9]. One reason for this phenomenon is that the libraries are typically screened using the same assay: a traditional growth-based assay. Two emerging approaches to improving the success of antifungal screening are (1) chemical genetic-based screens in which mutants of pathogenic fungi are used to increase the sensitivity and specificity of growth-based assays relative to wild-type pathogens and (2) application of non-growth-based assays with increased sensitivity and/or specificity [10], [11]. The former approach, the subject of an excellent recent review [9], has been highly successful in both the academic and industrial setting.

As an alternative to growth-based antifungal assays, our laboratory developed a high throughput assay that directly detects molecules that kill yeast cells using the release of the intracellular enzyme adenylate kinase (AK) as a reporter of cell lysis in *S. cerevisiae*, *Candida spp*., and *C. neoformans* [11]. Intracellular AK is released into the culture media when cellular integrity is disrupted and is then detected by a coupled reaction leading to luciferase-generated luminescence. The AK assay is specific for fungicidal molecules, giving no signal in the presence of a fungistatic agent such as fluconazole. In addition, the AK assay is more sensitive than growth-based assays and detects the activity of echinocandins such as caspofungin at concentrations 10-fold below the growth-based assay. This feature is particularly attractive for screening natural products libraries for low abundance molecules.

Using this assay, we screened libraries of off-patent drugs and identified 75 agents with fungilytic activity toward yeast, including the estrogen receptor antagonist tamoxifen (Figure 1D). The in vitro antifungal activity of tamoxifen was first reported in 1989 by Wiseman et al. but was not explored further [12]. Further work in our lab demonstrated that tamoxifen treatment decreases kidney fungal burden in a mouse model of candidiasis and provided evidence that at least part of its anti-fungal activity is due to inhibition of calmodulin [13]. The success of the chemical genetics approaches as well as the AK assay suggests that the application of creative new assay technologies to anti-infective discovery may allow the identification of new molecular scaffolds that went undetected by traditional, growth-based screening.

## New Fungal Cell Wall–Targeted Molecular Scaffolds

In recent years, one of the groups most active in antifungal drug discovery has been Merck. As summarized in an excellent recent review [9], Merck researchers identified a number of novel antifungal molecules and, importantly, characterized their cellular targets. An attractive antifungal drug target is the fungal cell wall because the structure is absent from host cells and, thus, molecules that inhibit its synthesis are likely to have low human toxicity. Although the bacterial cell wall is the target of a number of archetypal antibiotics (e.g., penicillin), only one class of antifungal drugs, the echinocandins, targets the fungal cell wall. Encouragingly, a number of new chemical scaffolds targeting the fungal cell wall have been reported in recent years. Indeed, Merck has reported a class of orally active inhibitors of β-1,3-glucan synthase [14], the same target as the intravenously administered echinocandins; the advantages of orally active drugs include better patient compliance, lower costs, and improved patient safety.

β-1,6-glucan is another crucial component of the fungal cell wall, and recently inhibitors of it synthesis (Figure 1E) have been reported to have in vitro activity against a range of *Candida* spp [15]. Like the β-1,3-glucan synthesis inhibitors reported to date, the β-1,6-glucan synthesis inhibitors have little or no in vitro activity against *C. neoformans*. In contrast, E1210 (Figure 1F), a novel, orally active isoxazole-based inhibitor of glycosylphosphatidylinositol (GPI)-linked protein biosynthesis, does have good activity against *C. neoformans* as well as a wide range of medically relevant yeasts and molds [16], [17]. Quite recently, a second chemotype of GPI-protein biosynthesis inhibitor has been identified [18]; interestingly, this molecule increases the immunogenicity of *C. albicans*, possibly by disrupting the mannoprotein outer layer of the cell wall and unmasking the more immunogenic inner β-glucan layer. GPI-linked mannoproteins are crucial components of the fungal cell wall, and identification of molecules that inhibit their production is an exciting development.

Finally, our laboratory used the AK assay in a screen of mechanistically distinct protein kinase inhibitors designed to identify fungilytic inhibitors of the cell wall integrity kinase signaling cascade, a stress response pathway conserved across pathogenic fungi. From this screen and subsequent structure-activity studies, we discovered that two distinct structural classes of human phosphoinositide-dependent kinase 1 (PDK1) inhibitors have potent antifungal activity and disrupt cell wall signaling [19]. hPDK1 is an important anti-cancer target because its inhibitors are well tolerated by normal mammalian cells and PDK1 inhibitors such as OSU-0312 (Figure 1G) have advanced to early phase clinical trials.

## Perspective

An interesting theme shared by the new antifungal targets described above is that many target proteins with orthologs in human cells. Since the targets of most current antifungal drugs are unique to fungi, this represents a significant conceptual evolution that seeks to exploit the sometimes subtle differences in protein structure between host and pathogen to identify molecules with selectivity for the fungal protein and, thereby, acceptable toxicity toward the host. The viability of this approach is due to powerful recent advances in structural biology and medicinal chemistry. Finally, it is important to emphasize that new developments in drug discovery should not replace older approaches but be additive and, thus, be used to expand the tool box of methods available for application to an increasingly important research problem.

## References

[1] Lai CC, Tan CK, Huang YT, Shao PL, Hsueh PR (2008) Current challenges in the management of invasive fungal infections. J Infect Chemother 14: 77–85. 18622668 10.1007/s10156-007-0595-7

[2] Park BJ, Wannemuehler KA, Marston BJ, Govender N, Pappas PG, et al (2009) Estimation of the current global burden of cryptococcal meningitis among persons living with HIV/AIDS. AIDS 23: 525–530. 19182676 10.1097/QAD.0b013e328322ffac

[3] Brown GD, Dennings DW, Levitz SM (2012) Tackling human fungal infections. Science 336: 647. 22582229 10.1126/science.1222236

[4] Blankenship JR, Steinbach WJ, Perfect JR, Heitman J (2003) Teaching old drugs new tricks: reincarnating immunosuppressants as antifungal drugs. Curr Opin Investig Drugs 4: 192–199. 12669381

[5] Cowen LE, Singh SD, Kohler JR, Collins C, Zaas AK, et al (2009) Harnessing Hsp90 function as a powerful, broadly effective therapeutic strategy for fungal infectious disease. Proc Natl Acad Sci U S A 106: 2818–2823. 19196973 10.1073/pnas.0813394106 PMC2650349

[6] Zhai B, Wu C, Wang L, Sachs MS, Lin X (2012) The antidepressant sertraline provides a promising therapeutic option for neurotropic cryptococcal infections. Antimicrob Agents Chemother 56: 3758–3766. 22508310 10.1128/AAC.00212-12 PMC3393448

[7] Breger J, Fuchs BB, Aperis G, Moy TI, Ausubel FM, et al (2007) Antifungal chemical compounds identified using a C. elegans pathogenicity assay. PLoS Pathog 3: e18 doi:10.1371/journal.ppat.0030018. 17274686 10.1371/journal.ppat.0030018 PMC1790726

[8] Spitzer M, Griffiths E, Blakely KM, Wildenhain J, Ejim L, et al (2011) Cross-species discovery of syncretic drug combinations that potentiate the antifungal fluconazole. Mol Syst Biol 7: 499. 21694716 10.1038/msb.2011.31 PMC3159983

[9] Roemer T, Xu D, Singh SB, Parish CA, Harris G, et al (2011) Confronting the challenges of natural product-based antifungal discovery. Chem Biol 18: 148–164. 21338914 10.1016/j.chembiol.2011.01.009

[10] Tebbets B, Stewart D, Lawry S, Nett J, Nantel A, et al (2012) Identification and characterization of antifungal compounds using a saccharomyces cerevisiae reporter bioassay. PLoS ONE 7: e36021 doi:10.1371/journal.pone.0036021. 22574132 10.1371/journal.pone.0036021 PMC3344848

[11] Krysan DJ, Didone L (2008) A high-throughput screening assay for small molecules that disrupt yeast cell integrity. J Biomol Screen 13: 657–664. 18626115 10.1177/1087057108320713

[12] Wiseman H, Cannon M, Arnstein HR (1989) Observation and significance of growth inhibition of Saccharomyces cerevisiae (A224A) by the anti-oestrogen drug tamoxifen. Biochem Soc Trans 17: 1038–1039. 2697611 10.1042/bst0171038

[13] Dolan K, Montgomery S, Buchheit B, Didone L, Wellington M, et al (2009) Antifungal activity of tamoxifen: in vitro and in vivo activities and mechanistic characterization. Antimicrob Agents Chemother 53: 3337–3346. 19487443 10.1128/AAC.01564-08 PMC2715577

[14] Walker SS, Xu Y, Triantafyllou I, Waldman MF, Mendrick C, et al (2011) Discovery of a novel class of orally active antifungal beta-1,3-D-glucan synthase inhibitors. Antimicrob Agents Chemother 55: 5099–5106. 21844320 10.1128/AAC.00432-11 PMC3195001

[15] Kitamura A, Someya K, Hata M, Nakajima R, Takemura M (2009) Discovery of a small-molecule inhibitor of {beta}-1,6-glucan synthesis. Antimicrob Agents Chemother 53: 670–677. 19015325 10.1128/AAC.00844-08 PMC2630612

[16] Miyazaki M, Horii T, Hata K, Watanabe NA, Nakamoto K, et al (2011) In vitro activity of E1210, a novel antifungal, against clinically important yeasts and molds. Antimicrob Agents Chemother 55: 4652–4658. 21825291 10.1128/AAC.00291-11 PMC3186989

[17] Hata K, Horii T, Miyazaki M, Watanabe NA, Okubo M, et al (2011) Efficacy of oral E1210, a new broad-spectrum antifungal with a novel mechanism of action, in murine models of candidiasis, aspergillosis, and fusariosis. Antimicrob Agents Chemother 55: 4543–4551. 21788462 10.1128/AAC.00366-11 PMC3187015

[18] McLellan CA, Whitesell L, King OD, Lancaster AK, Mazitschek R, et al (2012) Inhibiting GPI anchor biosynthesis in fungi stress the endoplasmic reticulum and enhances immunogencity. ACS Chem Biol. E-pub ahead of print doi.org/10.1021/cb300235m. 10.1021/cb300235m 22724584

[19] Baxter BK, DiDone L, Ogu D, Schor S, Krysan DJ (2011) Identification, in vitro activity and mode of action of phosphoinositide-dependent-1 kinase inhibitors as antifungal molecules. ACS Chem Biol 6: 502–510. 21294551 10.1021/cb100399x PMC3098953

